# SPHARM-PDM based image preprocessing pipeline for quantitative morphometric analysis (QMA) for in situ joint assessment in rabbit and rat models

**DOI:** 10.1038/s41598-021-04542-8

**Published:** 2022-01-21

**Authors:** Pholpat Durongbhan, Catherine E. Davey, Kathryn S. Stok

**Affiliations:** Department of Biomedical Engineering, The University of Melbourne, Parkville, VIC 3010 Australia

**Keywords:** Computer science, Musculoskeletal system, Data processing

## Abstract

The accessibility of quantitative measurements of joint morphometry depends on appropriate tibial alignment and volume of interest (VOI) selection of joint compartments; often a challenging and time-consuming manual task. In this work, we developed a novel automatic, efficient, and model-invariant image preprocessing pipeline that allows for highly reproducible 3D quantitative morphometric analysis (QMA) of the joint. The pipeline addresses the problem by deploying two modules: an alignment module and a subdivision module. Alignment is achieved by representing the tibia in its basic form using lower degree spherical harmonic basis functions and aligning using principal component analysis. The second module subdivides the joint into lateral and medial VOIs via a watershedding approach based on persistence homology. Multiple repeated micro-computed tomography scans of small (rat) and medium (rabbit) animal knees were processed using the pipeline to demonstrate model invariance. Existing QMA was performed to evaluate the pipeline’s ability to generate reproducible measurements. Intraclass correlation coefficient and mean-normalised root-mean-squared error of more than 0.75 and lower than 9.5%, respectively, were achieved for joint centre of mass, joint contact area under virtual loading, joint space width, and joint space volume. Processing time and technical requirements were reduced compared to manual processing in previous studies.

## Introduction

Recent advances in 3-dimensional (3D) image acquisition methods have allowed high-resolution medical images to be readily available, both clinically and preclinically^1^. This, coupled with an exponential increase in computing capability of modern devices, has led to rapid developments in the field of 3D quantitative morphometric analyses and has allowed researchers and clinicians to observe disease progression and evaluate their treatments with higher precision and sensitivity than before^2^. In the context of quantitative analysis of musculoskeletal tissues, many morphometric measures have been developed for computed tomography (CT)^3–6^ and magnetic resonance (MR)^7–10^ images to evaluate tissue and structural changes of the bone and cartilage. Recent studies^11,12^ have proposed a suite of 17 quantitative morphometric analysis measures (QMA) describing structures of the joint to assess it as a single organ and have demonstrated its reproducibility and sensitivity in assessing joint health in preclinical small and medium animal models using ex vivo micro-computed tomography (microCT) datasets of intact rat and rabbit knee joints. The QMA consisted of traditional structural measures of the bone and cartilage, as well as novel 3D whole joint measures (joint QMA) that include an angle to quantify osteophytes presence and activity (σ), an angle to indicate erosion between lateral and femoral condyles (ρ), a vector defining altered angulation (λ, α, β, γ), measures of joint space width (JSW), and a slope and intercept (m, χ) of joint contact area under virtual loading.

As discussed in earlier works^11,12^, one of the main issues surrounding joint QMA is that it is highly sensitive to the correct alignment of the joint to a common position, as well as the appropriate subdivision of the joint into its medial and lateral components for separate joint QMA measurements of each side. The common orientation is defined as the position where the long axis of the tibia is aligned with the vertical z-axis while the rest of the joint is rigidly transformed accordingly to preserve the original relative pose of all joint components. Previously, this was achieved by manually aligning one joint sample as a reference. All other samples were then registered and transformed onto that reference using B-spline interpolation^13^ to reduce rotational errors^14^. Selection of the volume of interest (VOI) of the joint’s medial and lateral sides was subsequently done manually by an expert through visual inspection of the 3D images according to appropriate anatomical features.

However, the manual nature of the initial alignment process, as well as the selection of the medial and lateral joint VOI has meant the quality of the joint QMA measurements is highly dependent on the skills and training of the operators to achieve high reproducibility and disease discriminating quality. Moreover, the diversity of animal models involved in preclinical OA research introduces a variety of imaging resolutions^15–18^. Consequently, settings for image processing operations such as dilation, erosion, closing, opening, and others, in the workflow must also be manually adjusted to achieve a reliable result. This is a non-trivial task that prohibits the use of a simple adaptation between models^19^, and is a time-consuming process that can occupy skilled operators for several weeks per study and present significant challenges to the robust application of joint QMA measurements in studies involving larger volumes of data and new joint models.

To tackle these challenges, this study presents an image processing pipeline that can automatically perform appropriate joint alignment and subdivision for efficient and high-quality 3D joint QMA while remaining robust across multiple small animal models. This study aims to estimate the shape and pose of the tibia and, by extension, the joint, based on a shape analysis technique called the spherical harmonic description method (SPHARM)^20^, which employs spherical harmonic descriptors in distinction to the traditional registration-based approach. Medial and lateral subdivision of the VOI is implemented by locating the dividing point using a watershedding approach based on persistent homology^21^, which is a topological data analysis method that identifies points with strong features. This study hypothesises that the pipeline will allow measurements of 3D joint QMA parameters with precision and reproducibility comparable to that of earlier studies that used manual alignment^11,12^, while achieving faster implementation and maintaining precision across species. Specifically, this study aims to compare measurement precision and reproducibility, as well as the overall processing time between the presented novel method and the manual processing approach on the same datasets which were published previously ^11,12^.

## Materials and methods

### Animals and microCT scan protocols

Datasets were obtained from previous studies of intact ex vivo joints from rat^12^ and rabbit^11^and are described in detail in the respective publications. The rat dataset consists of microCT scans (SCANCO Medical AG, Brüttisellen, Switzerland) of 21 tibio-femoral joints from 11 age-matched, Wistar rats. With a typical medial–lateral width of the tibia of 8.09 ± 0.62 mm, they were scanned with an isotropic nominal resolution of 10 µm. The rat dataset has an average image size in x, y, and z dimension of 1652.36 ± 221.39, 1416.59 ± 242, 943.5 ± 200.33 voxels and an average total number of voxels of 2.13 × 10^9^ ± 3.09 × 10^8^, respectively. The rabbit dataset consists of microCT scans of 6 tibio-femoral joints from 6 age-matched, New Zealand white rabbits. With a typical medial–lateral width of the tibia around 19.10 ± 0.59 mm, they were obtained with an isotropic voxel size of 18 μm, average image size in the x, y, and z dimension of 2017.00 ± 43.14, 2017.00 ± 43.14, 1891.83 ± 462.15 voxels and an average total number of voxels of 7.65 × 10^9^ ± 1.59 × 10^9^, respectively. During image acquisition of both datasets, initial joint positioning was controlled by placing a wedge (approx. 160° angle) behind the knee to control flexion–extension. The femoral condyles and the tibial plateau were included in the volume of interest, with the upper limit defined as the epiphyseal bone of the femur and the lower limit defined as the epiphyseal bone of the tibia (approx. 35–40 mm in rabbits and 12–15 mm in rats). Both datasets were filtered using a constrained 3D Gaussian filter (window, *σ* = 1.2, support, *s* = 1) after image reconstruction.

For each joint, four scans were performed. PRE scans were obtained by scanning joints without any contrast agent, while three repeat scans (labelled HEX1, HEX2, and HEX3) were obtained after a single intraarticular injection of SiO2-microbeads (0–20 μm diameter) (SWARCO Vestglass GmbH, Recklinghausen, Germany) to allow the visualisation of the cartilage. The three repeat scans were carried out to test for reproducibility with re-positioning between each scan.

### Preprocessing pipeline for joint QMA

**Figure 1:**
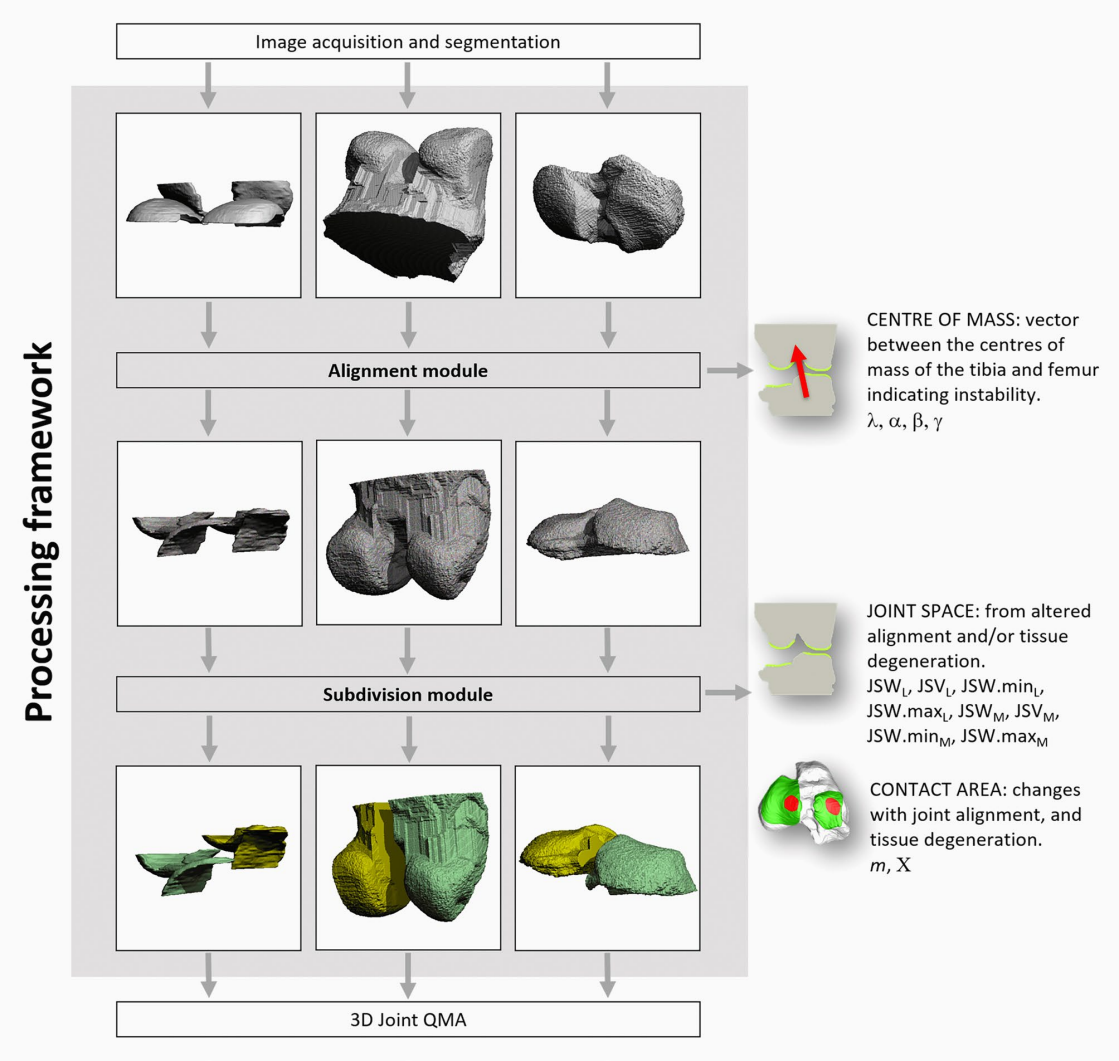
Overview of the pipeline as well as the relevant QMA used to evaluate each process. 3D microCT masks of cartilage (left column), femur (central column), and tibia (right column) of a typical rat knee is used to highlight each process’s input and result. Each of the outputs of the subdivision module (bottom row) is split into medial (yellow) and lateral (green) volume of interests.

The developed image preprocessing pipeline consists of 2 main components: an alignment module and a subdivision module. Filtered and segmented 3D binarised images of the femur, tibia, and their respective cartilage volumes are required inputs for the workflow. These images served as inputs for the alignment module. The resulting aligned, binarised images are used as inputs for the subdivision module which is the final module of the workflow. The images output from the pipeline are aligned and split into medial and lateral VOI and are ready for submission to the existing joint QMA analysis modules. An overview of the pipeline and the joint QMA measurements used to evaluate each process, are summarised in Fig. 1.

#### Alignment module

Joint alignment method in this work estimated the tibia’s elementary shape and pose using SPHARM^20^. In brief, SPHARM provides a hierarchical and multi-scale boundary description of objects with spherical topology. It computes an area-preserving mapping of the object’s 3D voxel mesh onto a sphere in a separate parameter domain. The basis functions of the sphere are spherical harmonics, so that SPHARM describes the object as a set of basis function weights. By truncating the number of basis functions used in the description, different levels of object detail can be represented.

The mathematics of SPHARM and the parameterisation computation according to Brechbühler^22^ employs Laplace’s spherical basis functions, ${Y}_{l}^{m}(\theta ,\varphi )$, characterised by degree, $l$ and order, $m$, for $\theta \in [0,\pi ]$ and $\varphi \in [\mathrm{0,2}\pi ]$, such that

1 $${Y}_{l}^{m}\left(\theta ,\varphi \right)= \sqrt{\frac{2l+1}{4\pi }\frac{\left(l-m\right)!}{\left(l+m\right)!}}{P}_{l}^{m}\mathrm{cos}\theta {e}^{im\varphi }$$

2 $${Y}_{l}^{-m}\left(\theta ,\varphi \right)={(-1)}^{m}{Y}_{l}^{m*}\left(\theta ,\varphi \right)$$

where ${Y}_{l}^{m*}(\theta ,\varphi )$ denotes the complex conjugate of ${Y}_{l}^{m}$ and ${P}_{l}^{m}$ describes the associated Legendre polynomials. Some of the low order real spherical harmonics as derived from the above equation are visualised in Supplementary Figure S1 online. This highlights the hierarchical representation of spherical objects where a higher spherical harmonics degree of $l$ would lead to more complex forms of $\theta$ and $\varphi$ and, thus, allows more details of the objects to be represented. Vice versa, by limiting the degree $l$ to lower degree, the object detail would be reduced, up to the point where the object is represented by a sphere at $l=0$.

To express the object’s surface using the described spherical harmonics, the three coordinate functions are decomposed and the surface ${\varvec{V}}\left(\theta ,\varphi \right)={(x\left(\theta ,\varphi \right),y\left(\theta ,\varphi \right),z\left(\theta ,\varphi \right))}^{T}$ takes the form of:

3 $${\varvec{V}}\left(\theta ,\varphi \right)= \sum_{l=0}^{\infty }\sum_{m=-l}^{l}{{\varvec{c}}}_{l}^{m}{Y}_{l}^{m}(\theta ,\varphi )$$

where the coefficients ${{\varvec{c}}}_{l}^{m}$ are 3D vectors of the three coordinates functions, obtained using a minimum squared error approach. Therefore, the values of the basis functions at each point in the discretised parameterised domain are gathered in the matrix, $z=\left({z}_{i,j\left(l,m\right)}\right)={Y}_{l}^{m}\left({\theta }_{i},{\varphi }_{i}\right)$ where $j(l,m)$ is a function assigning an index to every pair $\left(l,m\right)$ and $i$ denotes the indices of ${n}_{vert}$ points to be approximated. The coordinates of these points are arranged in the vector ${\varvec{v}}={({{\varvec{V}}}_{1},{{\varvec{V}}}_{2},\dots ,{{\varvec{V}}}_{{n}_{vert}})}^{T}$ and all coefficients are gathered in the vector ${\varvec{c}}={({{\varvec{c}}}_{0}^{0},{{\varvec{c}}}_{1}^{-1},{{\varvec{c}}}_{1}^{0},\dots )}^{T}$. The coefficients that best approximate the points from a least-square perspective are obtained by:

4 $${\varvec{c}}={({z}^{T}z)}^{-1}{z}^{T}{\varvec{v}}$$

**Figure 2:**
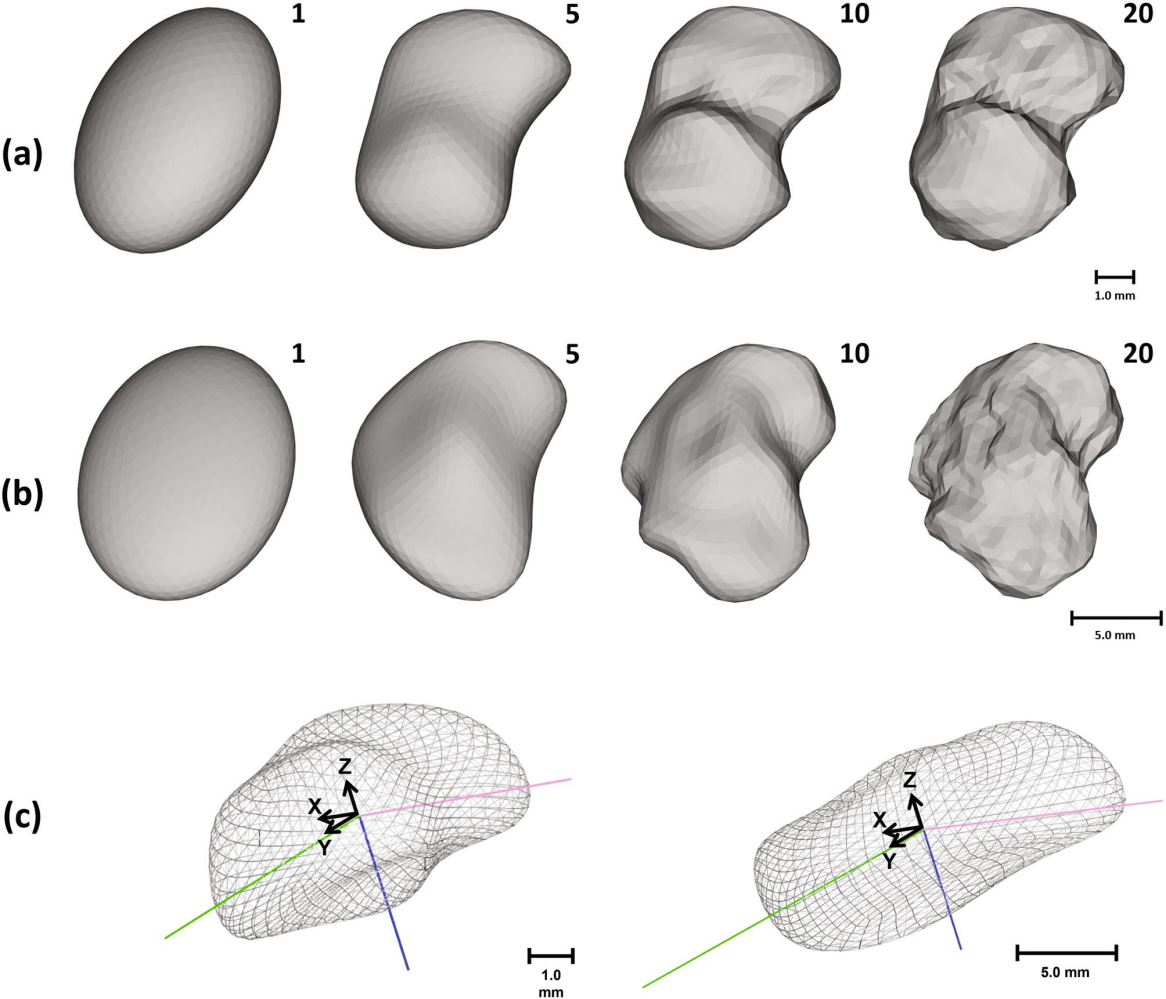
Isometric view of the SPHARM shape description of the proximal left tibial plateau of a typical rat (row a) and rabbit (row b) shown with different numbers of included basis functions of lowest degree (1, 5, 10, 20 harmonics, respectively). In row c, the shape of rat and rabbit tibia, represented by 5 harmonics and aligned towards a common orientation where the smallest principal component (blue) is aligned with the z-axis and the second smallest component (pink) is aligned with the x-axis are shown.

Using spherical harmonic basis functions, a hierarchical surface description is obtained that includes further details as more basis functions are added according to degree $l$ and order $m$ (see Fig. 2a,b).

After obtaining the tibia’s basic form from its truncated SPHARM description, principal component analysis (PCA) was performed. The smallest component of the tibia, which always aligns with its vertical axis, was used as the surrogate for the tibial vertical orientation, while the horizontal orientation was controlled by the second-smallest component. Subsequently, a series of transformation matrices aligning the smallest component with the z-axis and the second-smallest component with the x-axis was calculated, as seen in Fig. 2c, and was used to transform the full joint image using a B-spline interpolation method as the subsequent joint alignment step.

#### Subdivision module

**Figure 3:**
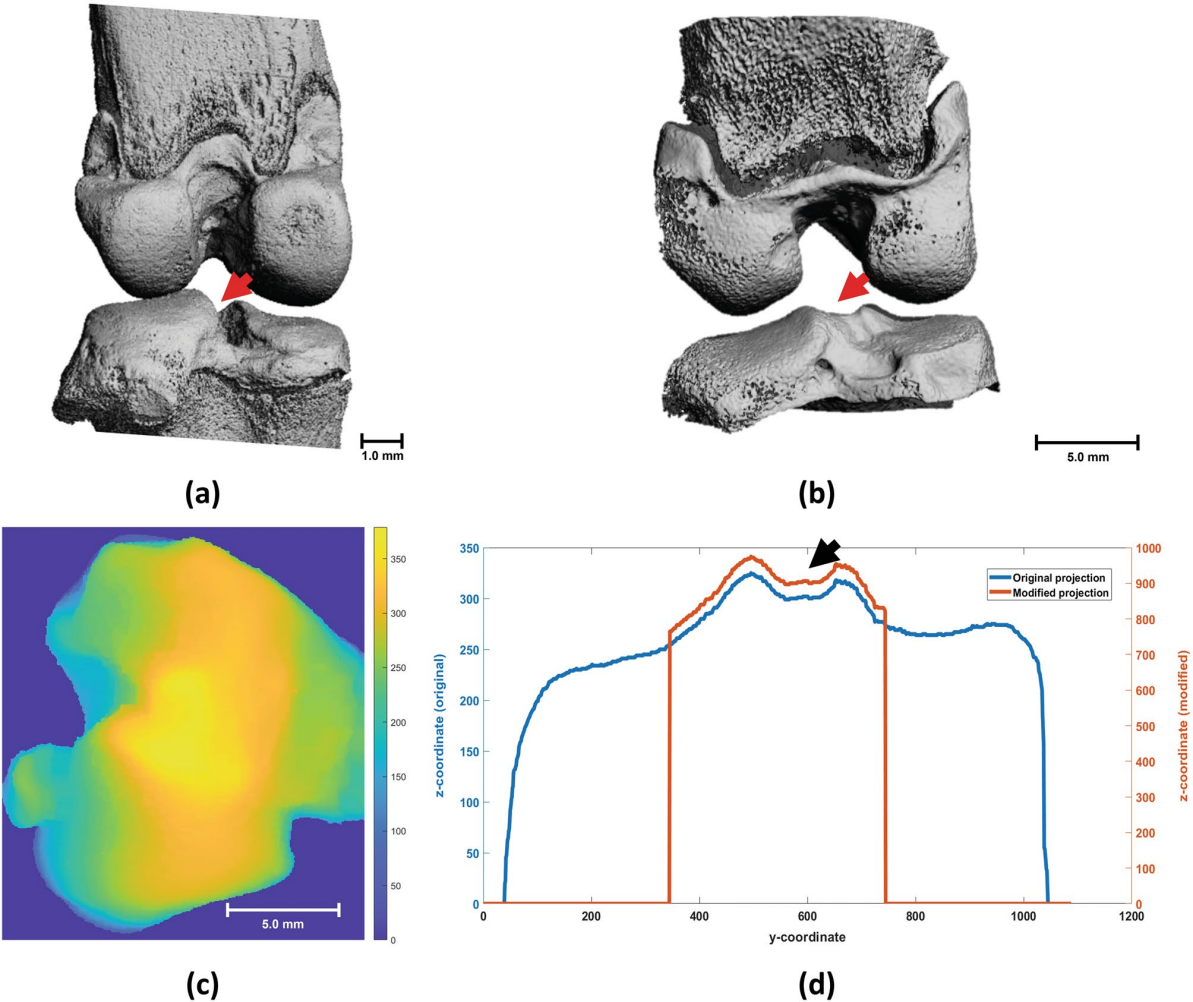
(**a**) Rat and (**b**) rabbit tibial intercondylar eminence used as the dividing point (red arrows) of the subdivision module. (**c**) Topographic map of the projections from a rabbit tibial plateau with values representing z-coordinates. (**d**) Subsequent 1D projection demonstrating the height profile of the tibia (blue line) as well as the cropped and boosted profile (orange line) used in actual computation of the point (black arrow).

Images aligned in the previous module were further processed through the subdivision module to divide the image of the joint into its medial and lateral VOI. The module operates by performing a watershedding operation based on persistence homology to identify the local minimum between the intercondylar tubercles of the tibia’s intercondylar eminence, as indicated in Fig. 3a,b, and using that location as the dividing point for defining the tibia, and subsequently the joint, medial and lateral side. The module achieves this by analysing a series of projections created from the aligned microCT dataset and taking advantage of the controlled alignment achieved in the previous module, where the axial and coronal planes are aligned with the z- and x-axis, respectively.

The subdivision module created a 2-dimensional (2D) topographic map of the tibial plateau’s features by projecting along the coronal plane as seen in Fig. 3c. Subsequently, the map was used to create a 1-dimensional (1D) projection on the coronal plane as illustrated by the blue profile in Fig. 3d. To further limit the impact of osteophytes on the dividing point detection algorithm, the 1D projection was cropped to exclude values not in the centre of the object which is the area where the intercondylar tubercle is located.

From the processed 1D projection map, the dividing point was located using persistence homology, which is an algebraic method for capturing topological features like local extrema^23,24^, computed using the **persistence1d**^25^ algorithm. In brief, the algorithm detects persistence homology from a 1D signal, which corresponds to all pairs of its local minima and maxima. Using this method, the global maxima is always paired with the background and has the highest persistence. The module’s watershedding approach removes all persistence pairs except the second-largest one, whose maxima corresponds to the minimum point between intercondylar eminences; and thus, defined as the watershed point used to divide and select the sub-images.

### 3D joint QMA, reproducibility, and accuracy

The joint centre of mass, defined as a vector with orientation (α, degree; β, degree; and γ, degree) measured along the three principle Cartesian axes, and connecting the centres of mass of the two bones, was used to measure the relative position of the joint to evaluate the performance of the alignment module^11^. To assess the subdivision module, as well as the pipeline as a whole, several 3D joint metrics were measured from the resulting medial (M) and lateral (L) sub-images of the joint. Joint contact area under virtual loading was also measured. This was done by incrementally shifting the tibia onto the femur along the vertical z-axis. The contact area is, then, defined as the distance travelled to first contact ($\chi_{L/M}$, μm) and the rate at which contact area increases ($m_{L/M}$, $\frac{{{\text{mm}}^{2} }}{step}$)^11^. Alongside these metrics, 3D measurements of the joint space width (JSW): joint space volume ($JSV_{L/M}$, ${\text{mm}}^{3}$), JSW ($JSW_{L/M}$, mm), minimum JSW ($JSW_{L/M} .min$, mm), and maximum JSW ($JSW_{L/M} .max$, mm) were also measured^12,26^.

Reproducibility of the measured 3D QMA was tested using RStudio (RStudio: Integrated development environment for R, Version 0.95.258, Boston, MA, USA). Precision error (PE(SD)) and precision error expressed as coefficients of variation (PE(%CV)), as well as an intraclass correlation coefficient (two-way random effects, consistency, multiple measurements ICC^27^) with 95% confidence interval (CI), were calculated to test the absolute agreement between each measurement^28^. ICC values range from 0 to 1, with those close to 1 indicating high similarity between measurements from the same group. Reproducibility of measurements is classified as excellent when ICC values are higher than 0.75^29^. Good, fair, and poor reproducibility is classified when ICC values range from 0.6 to 0.74, 0.40 to 0.59, and 0 to 0.4, respectively^29^.

Reproducibility results were compared with reproducibility values obtained in previous studies^11,12^ using the same dataset but has performed the tasks in this workflow manually. Additionally, to measure accuracy, the joint QMA results obtained in this study were compared with results from earlier studies^11,12^. Measurement differences were evaluated as root-mean-squared error expressed in both absolute (RMSE) and mean-normalised (NRMSE) form by using the original results as reference.

### Pipeline implementation and performance

The pipeline was implemented in two separate parts. SPHARM processing (statistical shape analysis module^30^ on 3DSlicer^31^), alignment determination, and dividing point location were done on Windows 10 running on Intel i7-10875H processor (8 cores, 16 MB cache, 2.30 GHz to 5.10 GHz) with Nvidia Quadro P620 GPU and 16 GB RAM, while the subsequent joint alignment and subdivision were done on networked OpenVMS V8.4 I64 running on HP Integrity rx2800 i2 platform (8 Intel Itanium CPUs, 1.6 GHz, 5 MB cache) with 32 GB of allocated RAM. The source code is available from the corresponding author upon request.

Pipeline performance was recorded as the average CPU time (in seconds) required to perform each process. Processing time was measured to enable benchmarking against manual processing time in earlier studies (in hours) on the same dataset^11,12^ whose estimates were obtained through examination of processing logs. Additionally, to benchmark the alignment module against another solution, the automatic joint alignment module was applied to the original images, without using SPHARM as part of the process.

## Results

To select the optimal SPHARM degree for the workflow, a parametric study where the ICCs of the resulting joint centre of mass were evaluated for an increasing number of harmonics. The results are available in Table S1 of the supplementary document which shows that a SPHARM degree of 5 yielded the highest ICC result and was used for all the subsequent results in this manuscript.

### Alignment module

For rat data, SPHARM-based module yielded excellent ICCs for all orientation measures (α: 0.955, β: 0.958, γ: 0.951) (Table 1) while alignment using full rat image yielded only good and fair ICCs for orientation measures (α: 0.508, β: 0.732, γ: 0.663) (Table 1). In contrast, alignment using full rabbit image yielded excellent and good ICCs (α: 0.965, β: 0.757, γ: 0.927) (Table 1), which were maintained or improved using SPHARM-based approach (α: 0.936, β: 0.920, γ: 0.934) (Table 1). For all centre of mass measurements in both models, minimal precision errors are reported with PE (%CV) lower than 2% for all values.

**Table 1:** Reproducibility of the rat and rabbit joint centre of mass in terms of intraclass correlation coefficient (ICC) and precision errors (PE) expressed in absolute and a percentage of the coefficient of variation of the repeated measure (α: angle with respect to x-axis, β: angle with respect to y-axis, γ: angle with respect to z-axis).

Rat (10 μm voxel size, 21 tibio-femoral joint samples, 4 repeated scans each)															
	Manually aligned^12^ using full image					Automatically aligned using full image					Automatically aligned using SPHARM descriptors				
	ICC	Lower 95%	Upper 95%	PE (SD)	PE (%CV)	ICC	Lower 95%	Upper 95%	PE (SD)	PE (%CV)	ICC	Lower 95%	Upper 95%	PE (SD)	PE (%CV)
α (°)	0.990	0.971	0.997	1.04	1.07%	0.508	0.207	0.800	21.90	34.96%	0.955	0.891	0.986	1.90	1.80%
β (°)	0.981	0.945	0.995	0.50	0.51%	0.732	0.483	0.906	4.54	5.21%	0.958	0.899	0.987	1.45	1.62%
γ (°)	0.998	0.995	0.999	0.35	0.47%	0.663	0.387	0.876	11.38	8.16%	0.951	0.884	0.985	1.67	1.06%

Rabbit (18 μm voxel size, 6 tibio-femoral joint samples, 4 repeated scans each)															
	Manually aligned^11^ using full image					Automatically aligned using full image					Automatically aligned using SPHARM descriptors				
	ICC	Lower 95%	Upper 95%	PE (SD)	PE (%CV)	ICC	Lower 95%	Upper 95%	PE (SD)	PE (%CV)	ICC	Lower 95%	Upper 95%	PE (SD)	PE (%CV)
α (°)	0.978	0.915	0.996	1.80	1.77%	0.965	0.884	0.994	4.95	9.24%	0.936	0.796	0.990	1.87	1.96%
β (°)	0.913	0.681	0.984	1.16	1.20%	0.757	0.408	0.955	5.55	6.22%	0.920	0.753	0.987	1.63	1.82%
γ (°)	0.981	0.931	0.996	1.46	0.92%	0.927	0.772	0.988	5.23	3.35%	0.934	0.790	0.989	1.29	0.76%

### Subdivision module

Excellent reproducibility was achieved for the contact area QMA measured from both rat and rabbit samples. Results for rat data showed slightly lower ICC for χ of both medial and lateral side (${\chi }_{L}$: 0.871, ${\chi }_{M}$: 0.875) compared to ICC for both $m$ (${m}_{L}$: 0.972, ${m}_{M}$: 0.982) (Table 2). For rabbit data, all except ${m}_{M}$ with ICC of 0.841, has ICC of more than 0.9 (Table 2). For all contact area measurements of both models, small precision errors are reported with PE(%CV) lower than 10% for all values.

**Table 2:** Reproducibility of the rat and rabbit joint contact area under virtual loading in terms of intraclass correlation coefficient (ICC) and precision errors (PE) expressed in absolute and a percentage of the coefficient of variation of the repeated measure ($\upchi$: distance travelled to first contact, $\mathrm{m}$: rate at which contact area increases).

Rat (10 μm voxel size, 21 tibio-femoral joint samples, 4 repeated scans each)										
	Manually subdivided^12^					Automatically subdivided				
	ICC	Lower 95%	Upper 95%	PE (SD)	PE (%CV)	ICC	Lower 95%	Upper 95%	PE (SD)	PE (%CV)
χ (mm)										
Lateral	0.957	0.877	0.988	0.01	4.27%	0.871	0.689	0.960	0.01	3.10%
Medial	0.925	0.777	0.980	0.01	5.57%	0.875	0.698	0.961	0.01	2.80%
*m* (mm^2^/mm)										
Lateral	0.966	0.902	0.991	1.04	3.00%	0.972	0.924	0.992	2.87	2.84%
Medial	0.992	0.976	0.998	0.42	1.30%	0.982	0.950	0.995	6.98	5.75%

Rabbit (18 μm voxel size, 6 tibio-femoral joint samples, 4 repeated scans each)										
	Manually subdivided^11^					Automatically subdivided				
	ICC	Lower 95%	Upper 95%	PE (SD)	PE (%CV)	ICC	Lower 95%	Upper 95%	PE (SD)	PE (%CV)
χ (mm)										
Lateral	0.888	0.547	0.979	0.04	9.58%	0.952	0.815	0.993	0.01	4.39%
Medial	0.754	0.202	0.951	0.03	8.72%	0.951	0.811	0.992	0.01	4.46%
*m* (mm^2^/mm)										
Lateral	0.980	0.928	0.996	1.11	5.26%	0.906	0.669	0.985	5.60	3.64%
Medial	0.903	0.642	0.982	0.95	4.18%	0.841	0.498	0.974	13.44	6.51%

For rat data, excellent reproducibility in was achieved for all joint space measurements, except $JSW.max$ where the reproducibility is very low (${JSW.max}_{L}$: 0.384, ${JSW.max}_{M}$: 0.536). Among the rest, ICCs for $JSW.min$ are slightly lower (${JSW.min}_{L}$: 0.859, ${JSW.min}_{M}$: 0.874) than others which have ICCs of more than 0.9, as shown in Table 3. Similarly, as seen in Table 4, excellent ICCs were also achieved for rabbit joint space measurements with values ranging from 0.780 (for ${JSW.min}_{M}$) to 0.955 (for ${JSV}_{L}$) except for ${JSW.max}_{L}$, which have an ICC of 0.608. Small precision error is also reported for both models, with PE(%CV) lower than 6.5% for all values except rabbit ${JSW.min}_{M}$ with PE(%CV) of 15.11%.

**Table 3:** Reproducibility of the rat and rabbit joint space measurements in terms of intraclass correlation coefficient (ICC) and precision errors (PE) expressed in absolute and a percentage of the coefficient of variation of the repeated measure (JSW: mean joint space width, JSV: joint space volume, JSW.min: minimum joint space width, JSW.max: maximum joint space width).

Rat (10 μm voxel size, 21 tibio-femoral joint samples, 4 repeated scans each)										
	Manually subdivided ^12^					Automatically subdivided				
	ICC	Lower 95%	Upper 95%	PE (SD)	PE (%CV)	ICC	Lower 95%	Upper 95%	PE (SD)	PE (%CV)
JSW (μm)										
Lateral	0.986	0.960	0.996	0.02	3.34%	0.946	0.871	0.983	0.01	0.86%
Medial	0.968	0.906	0.991	0.01	2.23%	0.943	0.866	0.982	0.01	2.26%
JSV (mm^3^)										
Lateral	0.986	0.962	0.996	0.05	2.58%	0.952	0.886	0.985	0.04	2.41%
Medial	0.983	0.955	0.995	0.03	1.79%	0.905	0.785	0.970	0.09	5.44%
JSW.min (μm)										
Lateral	0.859	0.623	0.958	0.04	18.73%	0.911	0.789	0.972	0.01	5.15%
Medial	0.874	0.663	0.963	0.03	24.70%	0.924	0.824	0.976	0.02	6.41%
JSW.max (μm)										
Lateral	− 0.030	− 0.230	0.353	0.01	0.59%	0.384	0.089	0.725	0.02	2.15%
Medial	0.763	0.378	0.929	0.01	1.00%	0.536	0.236	0.815	0.01	1.11%

Rabbit (18 μm voxel size, 6 tibio-femoral joint samples, 4 repeated scans each)										
	Manually subdivided ^11^					Automatically subdivided				
	ICC	Lower 95%	Upper 95%	PE (SD)	PE (%CV)	ICC	Lower 95%	Upper 95%	PE (SD)	PE (%CV)
JSW (μm)	3D JSW was not performed in earlier study for this dataset									
Lateral						0.907	0.719	0.985	0.05	2.61%
Medial						0.941	0.811	0.991	0.04	2.20%
JSV (mm^3^)										
Lateral						0.955	0.853	0.993	0.01	3.96%
Medial						0.907	0.717	0.985	0.01	4.17%
JSW.min (μm)										
Lateral						0.916	0.742	0.986	0.05	5.86%
Medial						0.780	0.447	0.960	0.13	15.11%
JSW.max (μm)										
Lateral						0.608	0.202	0.919	0.09	2.36%
Medial						0.790	0.466	0.962	0.16	4.44%

**Table 4:** 3D joint QMA results for rat and rabbit datasets obtained in previous studies through manual processing, and in this study using the proposed automatic workflow. Accuracy of the automatically processed rat and rabbit measurements are shown in terms of root-mean-squared error expressed in absolute (RMSE) and mean-normalised (NRMSE) form (JSW: mean joint space width, JSV: joint space volume, JSW.min: minimum joint space width, JSW.max: maximum joint space width, α: angle with respect to x-axis, β: angle with respect to y-axis, γ: angle with respect to z-axis, $\upchi$: distance travelled to first contact, $\mathrm{m}$: rate at which contact area increases).

Rat (10 μm voxel size, 21 tibio-femoral joint samples, 4 repeated scans each)						
	Manually processed^12^		Automatically processed		RMSE	NRMSE
	Mean	± SD	Mean	± SD		
JSW (μm)						
Lateral	578.0	33.0	534.6	19.3	49.7	8.53%
Medial	683.7	66.5	629.4	54.2	60.1	8.88%
JSV (mm^3^)						
Lateral	1.40	0.16	1.40	0.15	0.05	3.27%
Medial	1.76	0.26	1.76	0.28	0.05	2.86%
JSW.min (μm)						
Lateral	246.8	40.8	224.5	40.3	23.5	9.50%
Medial	382.7	74.7	362.7	74.5	21.3	5.63%
JSW.max (μm)						
Lateral	996.6	27.3	975.7	26.7	22.4	2.24%
Medial	1045.0	17.0	1026.6	15.2	20.2	1.93%
α (°)	94.3	11.0	97.6	8.8	8.0	8.39%
β (°)	85.9	2.5	87.6	7.0	6.6	7.69%
γ (°)	167.2	4.2	168.5	7.5	6.6	4.00%
χ (mm)						
Lateral	0.25	0.02	0.27	0.02	0.02	7.10%
Medial	0.26	0.02	0.28	0.02	0.02	7.92%
*m* (mm^2^/mm)						
Lateral	91.1	15.9	96.6	16.5	6.3	6.81%
Medial	115.9	43.8	124.9	46.9	10.0	8.69%

Rabbit (18 μm voxel size, 6 tibio-femoral joint samples, 4 repeated scans each)						
	Manually processed^11^		Automatically processed		RMSE	NRMSE
	Mean	± SD	Mean	± SD		
JSW (μm)						
Lateral	3D JSW was not performed in earlier study for this dataset		1889.1	147.6	3D JSW was not performed in earlier study for this dataset	
Medial			1953.3	172.7		
JSV (mm^3^)						
Lateral			39.62	6.92		
Medial			45.32	5.57		
JSW.min (μm)						
Lateral			806.8	171.6		
Medial			872.1	224.6		
JSW.max (μm)						
Lateral			3742.8	121.4		
Medial			3616.0	294.2		
α (°)	89.0	6.5	94.1	7.0	7.0	7.78%
β (°)	90.4	1.8	89.2	5.1	6.5	7.33%
γ (°)	171.1	3.4	171.9	4.9	6.5	3.81%
χ (mm)						
Lateral	0.48	0.08	0.52	0.08	0.04	7.93%
Medial	0.50	0.08	0.54	0.09	0.04	8.18%
*m* (mm^2^/mm)						
Lateral	51.2	6.4	55.1	6.9	4.3	8.31%
Medial	77.7	8.4	83.3	10.0	6.5	8.43%

### 3D joint QMA result and accuracy

For both datasets, all 3D joint QMA measurements have differences from the manual measures of less than 9.5% (Table 4). For rabbit data, 3D joint space measurements were not performed, so that accuracy results are available only for the rat dataset which have differences from the manual measures of less than 9.5% (Table 4). JSV and JSW.max have particularly low differences; less than 3.5% and less than 2.5%, respectively (Table 4).

### Pipeline performance

As seen in Table 5, the average time the pipeline used to finish processing a sample is shorter (rat: 693 s, rabbit: 2,112 s) than manual processing done on the same datasets in previous rat and rabbit studies (7,200 s for initial alignment and an additional 2,700 s for each rat sample and an additional 5,400 s for each rabbit sample). It is noted that a relatively high standard deviation was observed (rat: 212 s, rabbit: 276 s) due to networked connections. The most time-consuming process of the pipeline was the subsequent joint alignment using B-spline interpolation (rat: 248 ± 69 s, rabbit: 883 ± 261 s) and SPHARM processing (rat: 184 ± 77 s, rabbit: 1,019 ± 61 s) while the subdivision location was the fastest (rat: 19 ± 10 s, rabbit: 10 ± 3 s).

**Table 5:** CPU time for each process of the pipeline expressed in mean (± SD) seconds.

	Alignment module						Subdivision module				Framework		Manual processing from earlier studies^11,12^	
	SPHARM processing time (s)		Alignment determination time (s)		Subsequent joint alignment time (s)		Subdivision point location time (s)		Subsequent joint subdivision time (s)		Total processing time (s)		Total processing time (s)	
	Mean	± SD	Mean	± SD	Mean	± SD	Mean	± SD	Mean	± SD	Mean	± SD	Mean	± SD
Rat	184	77	27	4	248	69	19	10	215	100	693	212	9,900	1,100
Rabbit	1,019	61	27	15	883	261	10	3	172	64	2,112	276	12,600	1,300

Total time for manual processing performed in earlier studies is shown on the last column for comparison.

## Discussions

In this study, an image processing pipeline, that automatically prepares 3D joints images for sensitive and reproducible joint QMA measurements, does not require adaptation between animal models and is computationally efficient, was developed. Using SPHARM spherical harmonics modelling, the pipeline describes the tibia’s basic form and determines the transformation needed to align the joint. The second module subdivides the joint into medial and lateral compartments by locating the tibia’s intercondylar eminence as the watershed point using persistence homology to divide the VOI. The novelty of this work lies in the development of a new image processing pipeline that has been proven to prepare images efficiently and automatically for high-quality QMA, while remaining robust across two different preclinical animal models. This allows researchers to effectively perform 3D joint QMA in studies involving a larger number of samples, such as in longitudinal studies, with less expertise and a reduced time requirements compared to manual processing. Coupled with its robustness across rat and rabbit species, two common preclinical models for OA research^32^, the pipeline has the potential to make 3D joint QMA a more accessible technique.

The alignment and subdivision pipeline, used in conjunction with 3D joint QMA, could be applied to studies involving other musculoskeletal diseases beyond OA to reveal previously unknown quantitative changes to the joint. Moreover, although the proposed pipeline was developed and validated on microCT data, it should be noted that the workflow starts after segmentation. Consequently, if the segmented components of a joint are available with appropriate resolution (i.e., two segmented bone images for the joint centre of mass and joint space width measurements, and additional cartilage images for contact area under virtual loading), the framework could be applied to calculate 3D joint QMA for other imaging modalities (such as magnetic resonance imaging or CT), in addition to other joint sites.

The pipeline also utilises SPHARM in a novel application. From its original proposal^20^, SPHARM has been widely used in statistical shape modelling for analysis of many organs, while others have found applications in using SPHARM descriptors for efficient image rotation estimation to take advantage of its hierarchical representation property^37,38^. However, few works have taken advantage of SPHARM’s ability to represent objects in hierarchical levels of details in the object space for image processing purposes. The approach used in this work eliminated complex computation needed to perform image registration with $O({n}^{2})$ complexity^33^ and focused instead on finding a single solution that aligned the principal components to the desired pose with $O(n)$ complexity. This performance improvement can be highlighted in Table 5, where the average time needed to calculate the transformation matrix for both rat and rabbit dataset were approximately 27 s, while the B-spline image transformation performed in the subsequent joint alignment step took much longer to complete.

Moreover, the pipeline’s use of an efficient algorithm for subdivision—locating persistence homology pairs by Kozlov and Weinkauf^25^, which has $O(n\mathrm{log}n)$ complexity—allows for highly efficient processing overall. On average, the pipeline finished processing each sample in 693 s for rat and 2,112 s for rabbit, which is a great reduction from the 2,700 s for each rat and 5,400 s for each rabbit needed in earlier studies^11,12^ with most of the reduction coming from automation of the alignment. As shown in Table 5, the major difference in processing time between both animals can be traced back to the SPHARM modelling and subsequent joint alignment processes, where the average time needed for a rabbit sample is significantly higher than that of a rat. This is likely due to the differences in animal size and imaging scale (imaged volume and resolution) as described in the imaging protocol and resulted in rabbit images containing significantly more voxels than rat images (5.52 × 10^9^ more voxels on average). Therefore, more time was needed to perform SPHARM modelling and transformation. Other processes did not rely on the resolution of the images and resulted in similar processing times across animal models.

With regards to the quality of the QMA measurements, the pipeline is able to produce QMA measurement results with excellent reproducibility (ICC > 0.75) and precision errors (< 2% PE(%CV)) for the centre of mass and < 10% PE(%CV) for contact area but have comparable or slightly lower ICC compared to the gold-standard of manual processing by experts. As seen in Table 1, reproducibility values for the joint centre of mass from the pipeline are, generally, very slightly lower than that obtained from manually processed images from previous studies^11,12^. Reproducibility of contact area measurements, however, showed a mix of superior and inferior values when comparing with previous works. In general, manual processing in the earlier rat study^12^ produced slightly better ICC than those from the pipeline, while manual processing in the earlier rabbit study^11^ showed more mixed results as seen in Table 2. This could point to the increased experience of the operators in the follow-up study in the rat model^12^ as compared to the pioneering 3D joint QMA study of the rabbit model^11^.

It should be noted that the joint space measurements performed in the rat study^12^ and the rabbit study^11^ were not the same JSW modules. In the original QMA work^11^, JSW was measured as the distance of the joint space between the centre of the femoral condyle and the tibia; while, in the later work^12^, the JSW was directly measured in 3D using the SPECTRA consensus approach^26^ with JSV also being evaluated. In this study, the SPECTRA consensus approach was used to calculate JSW and JSV on both rat and rabbit dataset. As with the centre of mass and contact area measurements, reproducibility of the rat JSW and JSV showed excellent (ICC > 0.9), though slightly lower, values compared to results obtained with manual preprocessing. However, the pipeline results for ${JSW.min}_{M/L}$ have significantly lower precision error PE(%CV) as seen in Table 3. For rabbit data, this study also presents novel 3D JSW and JSV measurements with corresponding reproducibility values in Table 3 which shows excellent reproducibility for all joint space measures, except ${JSW.max}_{L}$ with ICC of 0.608. This generally high-quality result for previously unperformed joint space QMA for the rabbit dataset highlights the pipeline’s ability to correctly subdivide the images into the appropriate VOI and can support new QMA parameters in the future. With regards to low reproducibility of $JSW.max$, this study’s result affirms earlier study’s recommendation^12^ not to use in further analysis for rat knee. Moreover, the lower ICC for ${JSW.max}_{L}$ in rabbit sample also lead this study to recommend $JSW.max$ not be used for further analysis in rabbit knee as well.

The 3D joint QMA values measured using the proposed automatic workflow were shown to have similar values to those from earlier studies in Table 4. For both datasets, all measurements have errors of up to 9.5%, highlighting the sensitivity of 3D joint QMA measures to acquisition alignment. However, it should be further noted that there are no standard values of acceptable precision (PE(SD) and PE(%CV) and accuracy (RMSE and NRMSE) as they are highly dependent on the measurement context. Values of error for these measurements should be considered acceptable when the measurement approach provides results which are sensitive to the effects of disease or treatment, while maintaining consistent reproducibility values.

Since SPHARM modelling was used as the basis for its alignment algorithm, one of the essential requirements is that the input tibia must be spherical in nature. This presents one of the main limitations of the pipeline as any samples such as those bone with convex surfaces at the edge of the image due to the VOI cropping through the medullary canal will not meet this requirement and will fail during the SPHARM processing step. Some manual closing, and smoothing operations will have to be performed on these samples prior to being processed through the pipeline.

Additionally, even though the pipeline has solved the issue of joint alignment in the image processing stage, it is clear that systematic positioning of the joint during the acquisition of the images is fundamental to ensure that all joints are captured in a similar pose. In joint imaging where multiple rigid structures are of interest, it is not possible to simultaneously align both the femur and tibia without altering their original relative position which provides important pathological information^34^. Similar challenges have been noted in clinical measurement of radiological JSW, where reproducible patient position is required for reliable tracking of joint changes ^35,36^. Future improvements in this direction would be in the form of a standardised positioning for acquisition that would allow the joints to be scanned and rescanned with minimal difference in pose between each. Further investigation, in strict quantitative terms, the definition of a gold standard alignment from which joint QMA can be precisely measured should also be done. Together, it is expected that even higher measurement reproducibility could be achieved.

Moreover, further in vivo experiments with regards to sensitivity and reproducibility of the proposed workflow and the novel 3D joint QMA parameters is required to determine whether these ex vivo results can be replicated in vivo. Movement artifact in live animal may reduce the precision and accuracy of these measurements, requiring further development of the workflow.

## Conclusion

In previous work, quantitative measurements of joint morphometry using QMA has shown potential as a platform to quantify disease-based morphometric features for joint research using multiple preclinical animal models. However, its accessibility and usage are limited by high sensitivity to alignment and joint subdivision which are technically challenging and time-consuming to implement manually. In this work, we developed an automatic, efficient, and model-invariant image processing pipeline. The software was found to allow 3D joint QMA measurements with excellent reproducibility comparable to those obtained from manual processing in earlier studies.

## Supplementary Information

Supplementary Information.

## Data Availability

Datasets and all source code generated, used, and/or analysed during the current study are available from the corresponding author on reasonable request.
