# Supplementary material for: SPHARM-PDM based image preprocessing pipeline for quantitative morphometric analysis (QMA) for in situ joint assessment in rabbit and rat models
Source: Sci Rep. 2022 Jan 21;12:1113. doi: 10.1038/s41598-021-04542-8 (PMC8782854; doi:10.1038/s41598-021-04542-8)
Supplement: Supplementary file 1 — Supplementary Information. [file 41598_2021_4542_MOESM1_ESM.docx]

**SPHARM-PDM based image preprocessing pipeline for quantitative morphometric analysis (QMA) for *in situ* joint assessment in rabbit and rat models**

Pholpat Durongbhan¹, Catherine E. Davey¹, Kathryn S. Stok¹^*^

¹Department of Biomedical Engineering, University of Melbourne, Parkville, Australia

**Supplementary material**

**Supplementary figures**

*Supplementary Figure S1: Visual representations of real spherical harmonics for the first few low values of l and m.*


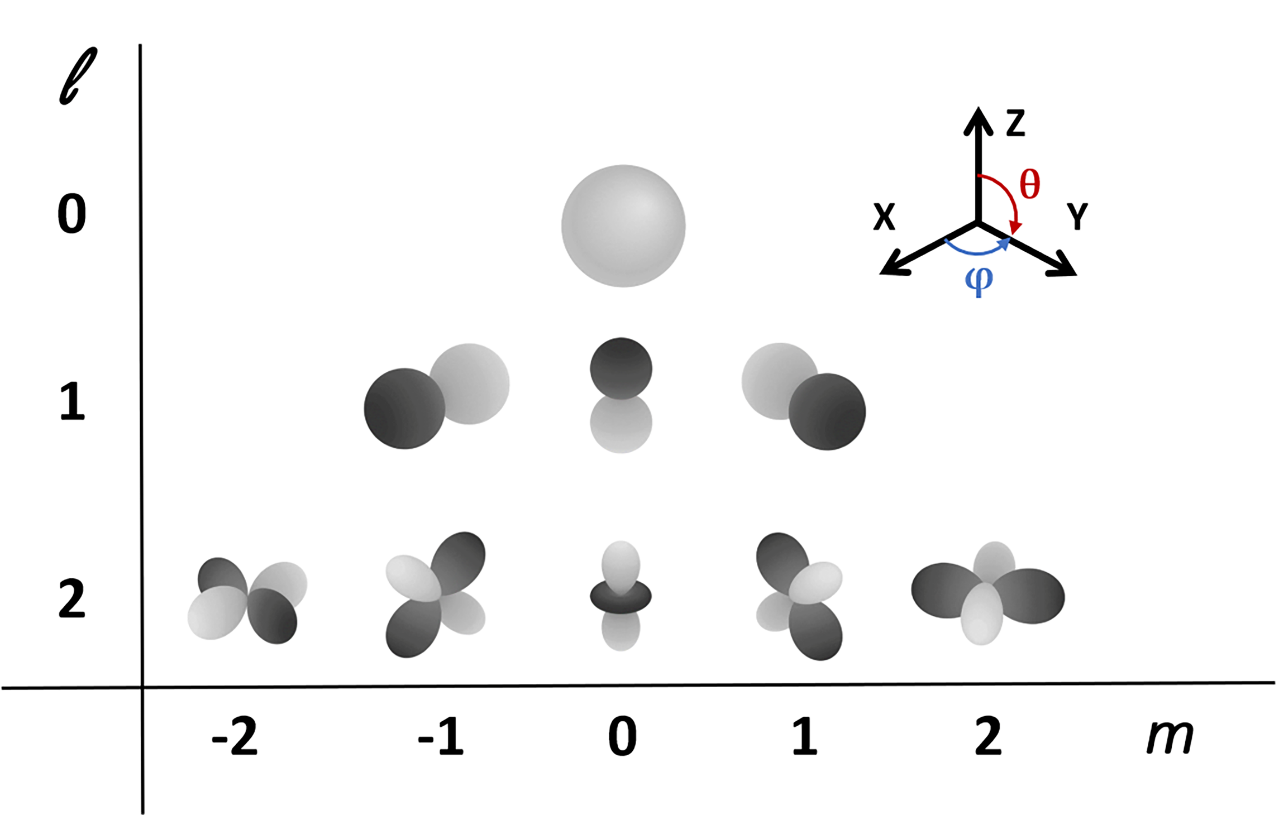


*Supplementary Figure S2: Sample-wise scatterplot of centre of mass measurement results for (a) rat and (b) rabbit samples after processing with the alignment module. Colour denotes repeated measurement of the same sample.*


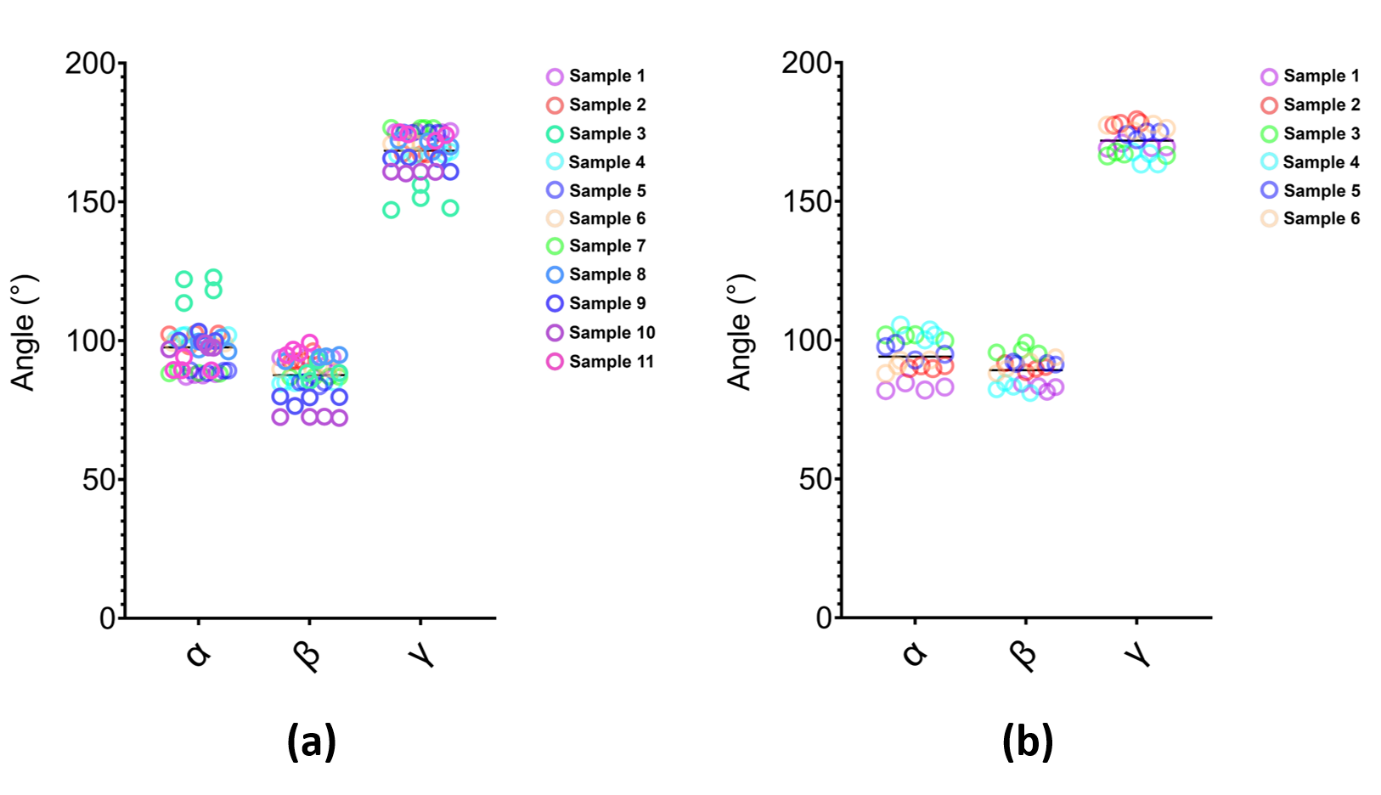


*Supplementary Figure S3: Sample-wise scatterplot of in silico loading contact area measurement results for rat (in row a) and rabbit (in row b) samples after processing with the alignment and subdivision module. Colour denotes repeated measurement of the same sample.*


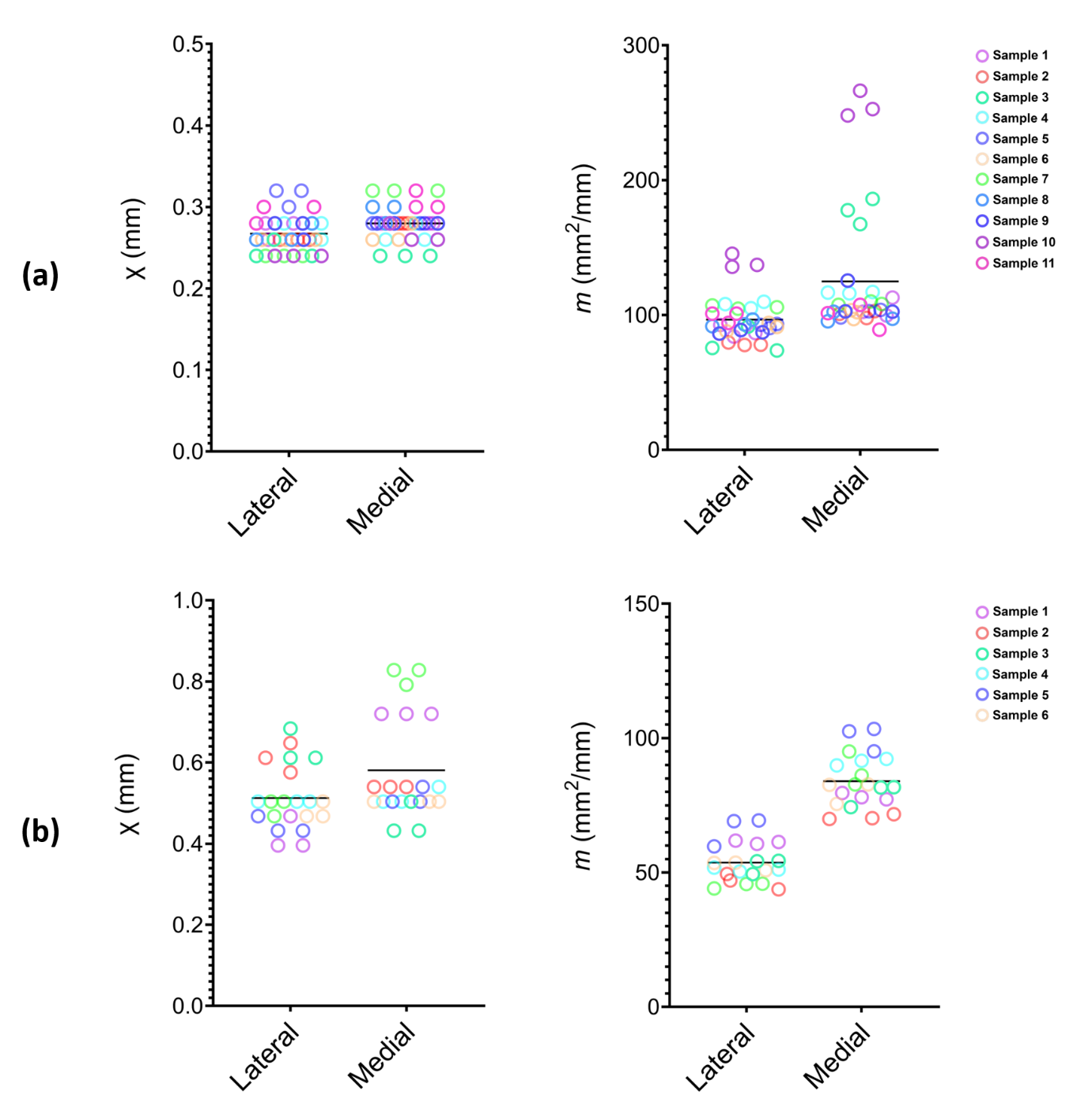


*Supplementary Figure S4: Sample-wise scatterplot of joint space measurement results for rat samples for (a) JSW and (b) JSV after processing with the alignment and subdivision module. Colour denotes repeated measurement of the same sample.*


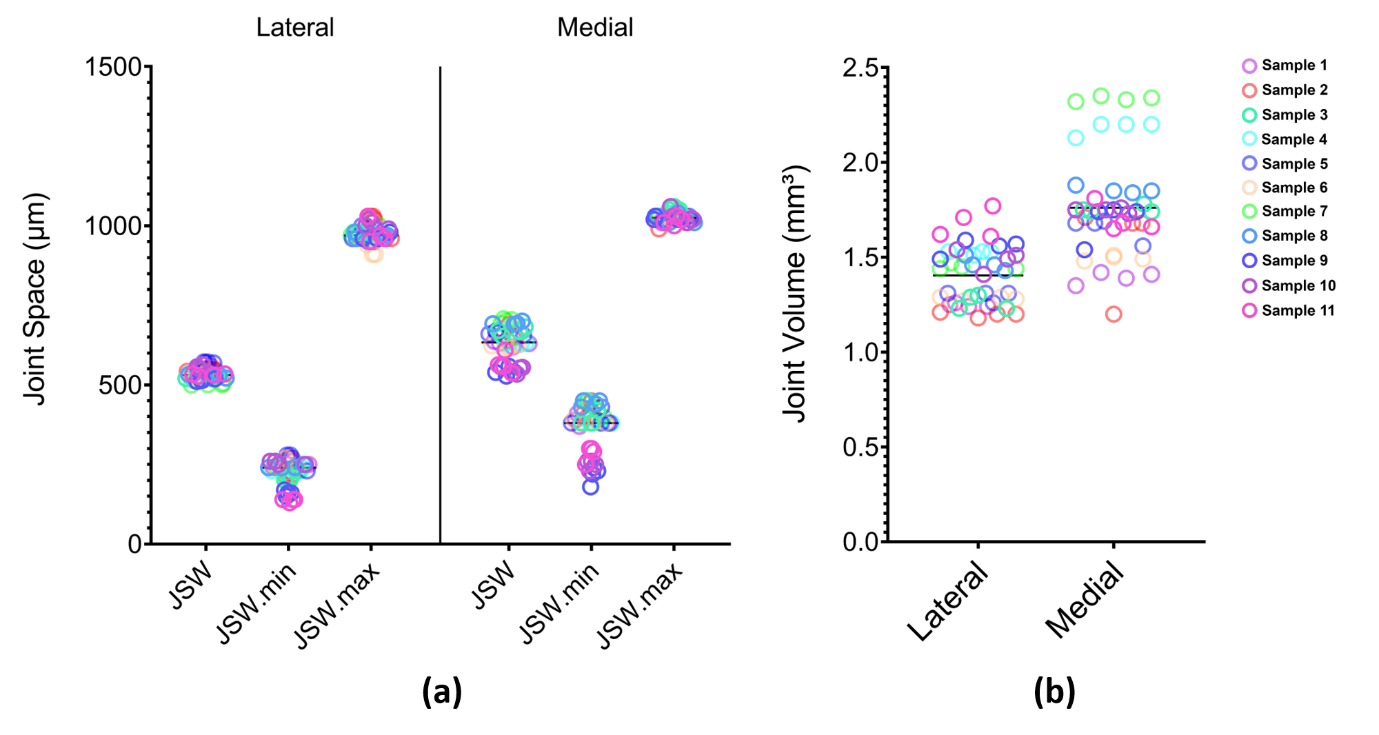


*Supplementary Figure S5: Sample-wise scatterplot of joint space measurement results for rat samples for (a) JSW and (b) JSV after processing with the alignment and subdivision module. Colour denotes repeated measurement of the same sample.*


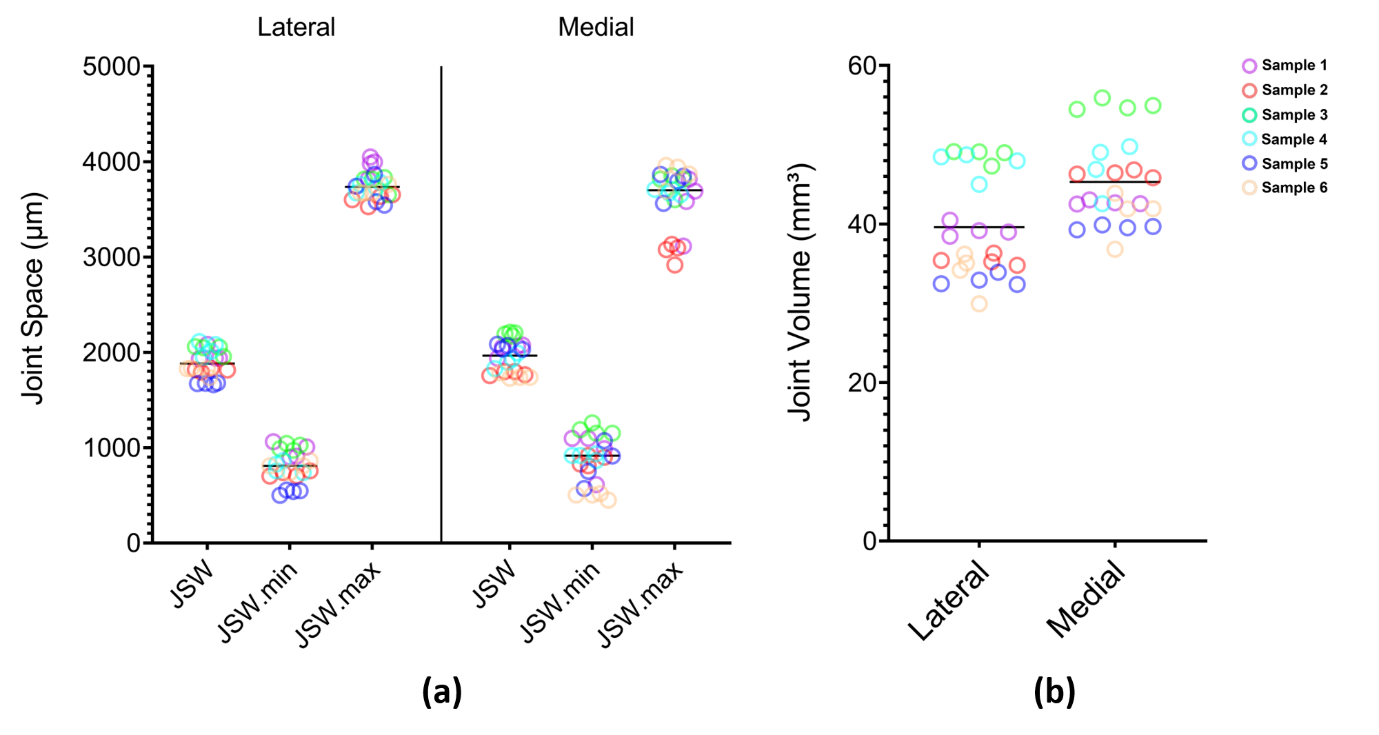


**Result of SPHARM degree parametric study**

*Supplementary Table S6: Reproducibility of the rat and rabbit joint centre of mass in terms of intraclass correlation coefficient (ICC) obtained by aligning models with different SPHARM degree. Gray row highlighted the most optimum SPHARM degree.*

|  | Rat (10 μm voxel size) | | | Rabbit (18 μm voxel size) | | |
| --- | --- | --- | --- | --- | --- | --- |
| SPHARM Degree | α | β | γ | α | β | γ |
| 20 | 0.954 | 0.956 | 0.952 | 0.942 | 0.915 | 0.915 |
| 10 | 0.953 | 0.955 | 0.950 | 0.940 | 0.919 | 0.915 |
| 5 | 0.955 | 0.958 | 0.951 | 0.936 | 0.920 | 0.934 |
| 4 | 0.954 | 0.954 | 0.951 | 0.938 | 0.920 | 0.915 |
| 3 | 0.954 | 0.955 | 0.952 | 0.938 | 0.919 | 0.915 |
| 2 | 0.956 | 0.954 | 0.952 | 0.940 | 0.920 | 0.916 |
| 1 | 0.955 | 0.953 | 0.951 | 0.938 | 0.919 | 0.916 |

**Reproducibility for 3D joint centre of mass of unprocessed data**

*Supplementary Table S7: Reproducibility of the rat and rabbit joint centre of mass in terms of intraclass correlation coefficient (ICC) and precision errors (PE) when PCA alignment was performed directly on unprocessed data.*

| Rat (10 μm voxel size) | | | | | |
| --- | --- | --- | --- | --- | --- |
| Unprocessed | | | | | |
|  | ICC | Lower 95% | Upper 95 % | PE (SD) | PE (%CV) |
| λ (mm) | 0.863 | 0.702 | 0.955 | 0.06 | 1.18% |
| α (°) | 0.577 | 0.282 | 0.836 | 10.98 | 13.60% |
| β (°) | 0.890 | 0.755 | 0.965 | 1.39 | 1.54% |
| γ (°) | 0.731 | 0.483 | 0.906 | 3.84 | 26.27% |
| Rabbit (18 μm voxel size) | | | | | |
| Unprocessed | | | | | |
|  | ICC | Lower 95% | Upper 95 % | PE (SD) | PE (%CV) |
| λ (mm) | 0.986 | 0.952 | 0.998 | 0.23 | 1.61% |
| α (°) | 0.901 | 0.702 | 0.984 | 2.38 | 2.71% |
| β (°) | 0.739 | 0.379 | 0.951 | 3.66 | 4.11% |
| γ (°) | 0.385 | -0.005 | 0.843 | 2.60 | 1.52% |
